# NoiBic: a noise-tolerant biclustering algorithm for high-throughput gene expression data analysis

**DOI:** 10.1093/bioinformatics/btag743

**Published:** 2026-10-07

**Authors:** Chaoyi Long, Jingxian Li, Bingqiang Liu, Duanchen Sun, Guojun Li

**Affiliations:** Research Center for Mathematics and Interdisciplinary Sciences (Ministry of Education Frontiers Science Center for Nonlinear Expectations), Shandong University, Qingdao, China; School of Mathematics, Shandong University, Jinan, China; School of Mathematics, Shandong University, Jinan, China; School of Mathematics, Shandong University, Jinan, China; Shandong Key Laboratory of Cancer Digital Medicine, Jinan, China; Research Center for Mathematics and Interdisciplinary Sciences (Ministry of Education Frontiers Science Center for Nonlinear Expectations), Shandong University, Qingdao, China

## Abstract

**Motivation:**

High-throughput transcriptomic technologies generate large-scale gene expression datasets for functional module discovery. However, technical and biological noise can obscure coherent expression patterns and compromise module detection. Noise-tolerant methods are therefore needed to recover meaningful biclusters.

**Results:**

We developed NoiBic, a noise-tolerant biclustering algorithm for high-throughput transcriptomic data. NoiBic integrates a longest approximate common subsequence-based seed identification strategy with noise-aware column expansion to improve bicluster detection under noisy conditions. Across simulated datasets with controlled settings for bicluster coherency, matrix size, bicluster number, noise level, and overlap level, NoiBic accurately recovered planted biclusters, particularly under noisy and overlapping conditions. Evaluation on bulk and single-cell RNA-seq datasets demonstrated its ability to identify biologically meaningful gene modules and cell type-associated biclusters.

**Availability:**

NoiBic is implemented in C++17 and available at https://github.com/lcyi0208/NoiBic. The software version used in this study has been permanently archived on Zenodo at https://doi.org/10.5281/zenodo.22732377. Benchmark datasets are available at https://doi.org/10.5281/zenodo.21919392.

## 1 Introduction

Large-scale transcriptome profiling enables the discovery of co-expression modules, groups of genes with shared expression patterns and functional or regulatory roles (Wang *et al.* 2009, Ozsolak and Milos 2011, Saelens *et al.* 2018). Because transcriptional regulation is context dependent, modules active in specific samples or cells are particularly informative. However, technical and biological noise in high-throughput expression data can obscure coherent patterns and compromise module detection (Fleming *et al.* 2023).

Biclustering addresses this challenge by simultaneously identifying subsets of genes and samples or conditions within an expression matrix (Ulitsky *et al.* 2010, Xie *et al.* 2019). Unlike conventional global co-expression network approaches, which primarily define gene modules from gene-gene relationships across samples, biclustering directly returns both module genes and the conditions in which they are active, making it well suited to discovering context-associated modules in heterogeneous transcriptomic data (Castanho *et al.* 2024).

Existing biclustering methods remain limited by noise tolerance or computational cost (Mills and Gordon 2001, Padilha and Campello 2017, Fleming *et al.* 2023). QUBIC2 uses left-truncated mixture Gaussian (LTMG)-based discretization to handle low expression values (Wan *et al.* 2019, Xie *et al.* 2020), whereas RecBic (Liu *et al.* 2020) and ARBic (Liu *et al.* 2023) relax the trend-preserving constraints to improve noise tolerance; however, these approaches are computationally demanding or sensitive to parameter settings.

Here, we propose NoiBic, a noise-aware biclustering framework for high-throughput transcriptomic data. Building on UniBic’s longest common subsequence (LCS)-based seed strategy, NoiBic introduces three main components: (i) a longest approximate common subsequence (LACS) algorithm; (ii) interval-based column expansion; and (iii) modeling of both concordant and discordant expression trends. Together, these components enable efficient identification of context-associated gene modules from noisy transcriptomic data.

## 2 Materials and methods

### 2.1 Overview of NoiBic

The NoiBic workflow is summarized in Fig. 1. After preprocessing, NoiBic performs seed selection, bicluster identification, expansion, and pruning. Candidate seeds are identified using LACS on row pairs, then expanded into approximately trend-preserving biclusters through noise-aware row and column expansion. Redundant overlaps are pruned from biclusters exceeding a user-defined overlap threshold. Bicluster pattern classes are illustrated in Supplementary Fig. S1, available as supplementary data at *Bioinformatics* online.

**Figure 1:**
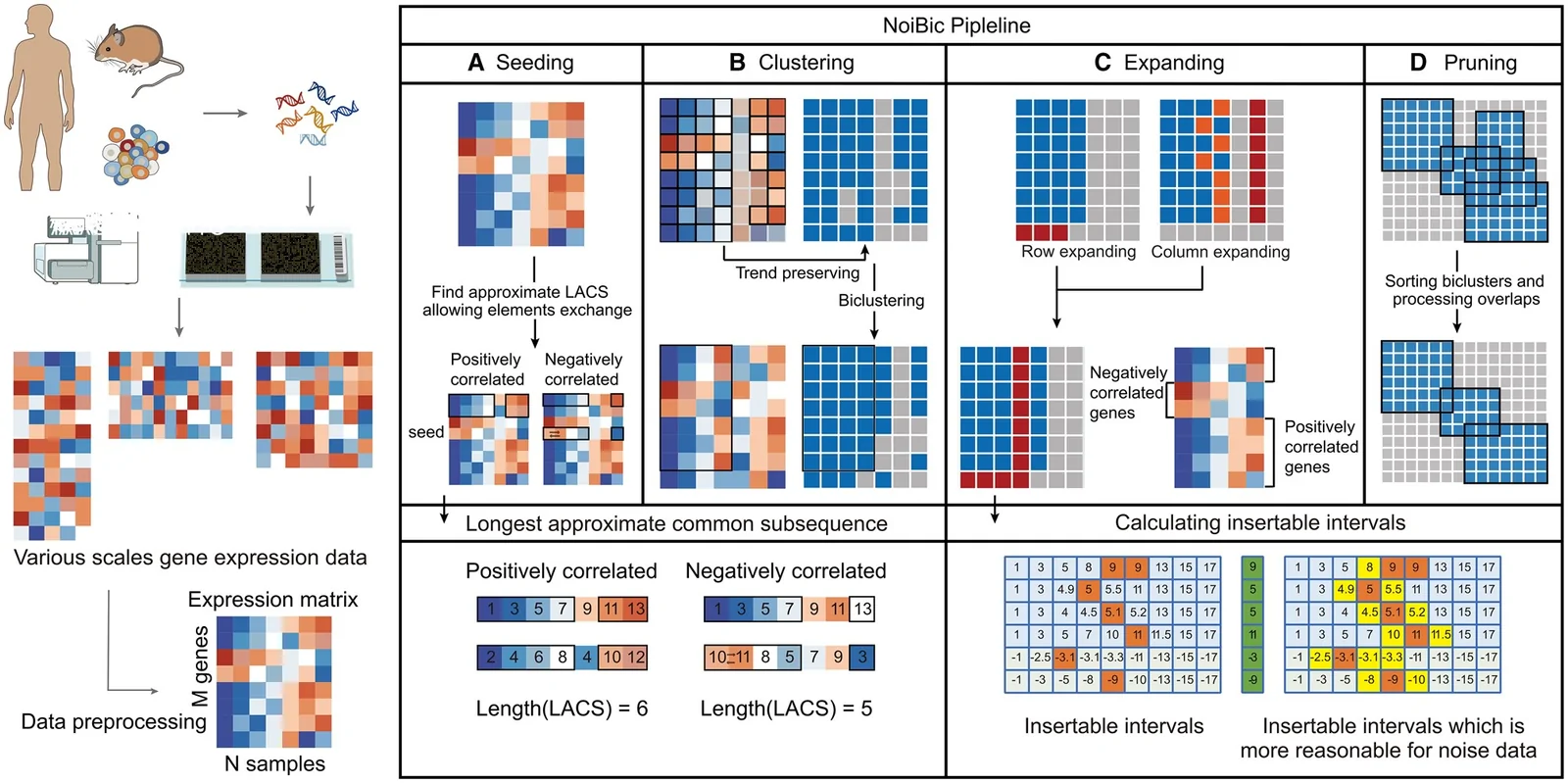
Overview of the NoiBic algorithm. (A) Seed selection via LACS algorithm on the sorted index matrix derived from normalized expression data; (B) iterative construction of trend-preserving submatrices by adding rows and pruning columns; (C) noise-aware row/column expansion; (D) pruning of overlapping biclusters to reduce redundancy.

### 2.2 Longest common subsequence algorithm

In UniBic, LCS is used to identify trend-preserving submatrices within an input matrix $M\in R^{n\times m}$. For two sequences $X=\{x_{1},x_{2},\ldots ,x_{i}\}$ and $Y=\{y_{1},y_{2},\ldots ,y_{j}\}$, $C_{i,j}$ denotes the LCS length and is computed as:


$$C_{i,j}=\{\begin{array}{c} 0,\;if\;i=0\;or\;j=0,\\ C_{i-1,j-1}+1,\;\;\;\;\;if\;x_{i}=y_{j},\\ \max\left(C_{i-1,j},C_{i,j-1}\right),\;if\;x_{i}\neq y_{j}. \end{array}$$

### 2.3 Longest approximate common subsequence algorithm

Since the classical LCS algorithm is sensitive to noise-induced rank fluctuations, we extend it to the longest approximate common subsequence (LACS) algorithm, which permits controlled exchanges between similar nearby elements. For LACS preprocessing, each row of M is sorted in ascending order to obtain the row-sorted matrix $M^{\prime }$ and the corresponding index matrix A, where $A_{ij}$ records the original column index of the j-th entry in $M_{i\cdot }^{\prime }$, such that


$$M_{iA_{ij}}=M_{ij}^{\prime }.$$

For any two rows u and v of A, LACS algorithm takes $A_{u\cdot }$ and $A_{v\cdot }$ as input sequences. Let $C_{i,j}\;$denote the LACS length between $\left\{A_{u,1},\;A_{u,2},\;\ldots ,\;A_{u,i}\right\}$ and $\left\{A_{v,1},\;A_{v,2},\;\ldots ,\;A_{v,j}\right\}$, recursively defined as


$$C_{i,j}=\{\begin{array}{cc} 0, & if\;i=0\;or\;j=0,\\ C_{i-1,j-1}+1, & if\;A_{u,i}\approx^{*}\;A_{v,j},\\ \max\left(C_{i,j-1},\;C_{i-1,j}\right), & \mathrm{otherwise}. \end{array}$$

Here, $\approx^{*}$ denotes approximate equality under the LACS criterion, satisfied when at least one of the following conditions holds:

1.
$A_{u,i}=A_{v,j^{\prime }}$

2. An exchange is performed between${\;A}_{u,i}$ and${\;A}_{u,i^{\prime }}$, where $i^{\prime }$ denotes the rank position in $A_{u\cdot }$ corresponding to $A_{v,j}$, such that $A_{u,i^{\prime }}=A_{v,j}$
3. An exchange is performed between${\;A}_{v,j}$ and $A_{v,j^{\prime }}$, where $j^{\prime }$ denotes the rank position in $A_{v\cdot }$ corresponding to $A_{u,i}$, such that $A_{v,j^{\prime }}=A_{u,i}$

For a given row u, the elements at positions i and $i^{\prime }$ in $M^{\prime }$, together with their original values, yield four quantities. An exchange between $A_{u,i}$ and $A_{u,i^{\prime }}$ is permitted only when $i^{\prime }>i$ and $j^{\prime }>j$, and when all pairwise combinations among these four values satisfy the following condition:


$$\frac{\left|m_{p}-m_{q}\right|}{\min\left(\left|m_{p}|,|m_{q}\right|\right)}\;\leq \;CV_{u}\cdot \alpha ,$$

where $m_{p}$ and $m_{q}$ denote any two of the four values, α is a user-defined tolerance parameter (default value is 0.1) controlling exchange strictness, and $CV_{u}$ denotes the coefficient of variation of row u. The same condition is symmetrically applied to row v. This constraint ensures that exchanged elements and their original values are sufficiently similar, maintaining local consistency within each sequence. Thus, LACS preserves approximate trend-preserving structure and reduces the influence of noise on bicluster identification. When intra‑row exchanges are admissible in both rows u and v, the algorithm computes row-specific exchange costs and performs the lower-cost swap. The exchange costs for row u and v are:


$$\mathrm{scor}e_{u}=\frac{\left|M_{u,i}^{\prime }-M_{u,i^{\prime }}^{\prime }\right|}{\min\left(\left|M_{u,i}^{\prime }|,|M_{u,i^{\prime }}^{\prime }\right|\right)\cdot CV_{u}},$$

$$\mathrm{scor}e_{v}=\frac{\left|{M^{\prime }}_{v,j}-M_{v,j^{\prime }}^{\prime }\right|}{\min\left(\left|{M^{\prime }}_{v,j}|,|M_{v,j^{\prime }}^{\prime }\right|\right)\cdot CV_{v}}.$$

Finally, the value of $C_{n,n}$ obtained after recursive iteration represents the LACS length between sequences $A_{u\cdot }$ and $A_{v\cdot }$. An illustrative comparison between LCS and LACS is provided in Supplementary Fig. S2, available as supplementary data at *Bioinformatics* online.

### 2.4 Seed selection

The index matrix A is randomly partitioned by rows into four groups, and LACS is computed for all row pairs within each group. For each row pair ($A_{u\cdot },\;A_{v\cdot })$, two LACS lengths are computed: one between $A_{u\cdot }$ and $A_{v\cdot }$, and the other between $A_{u\cdot }$ and the index sequence obtained by sorting row  v of M in descending order, reflecting potential opposite expression patterns between the two genes. The larger value is taken as the final LACS between rows u and v. If it exceeds the predefined threshold, the corresponding row pair is recorded as a candidate seed. All candidate seeds are ranked by decreasing LACS length. The top-k seeds are selected sequentially from this ranked list. By default, k is set to 20 times the user-specified number of output biclusters. A candidate seed is retained only if it satisfies two constraints. First, neither of its two rows can have appeared in the already selected seeds more than the predefined frequency threshold p, which is set to 3 by default. Second, relative to each previously selected seed, at least one row in the candidate seed must be separated from the rows of that selected seed by more than n/k in terms of row-index distance. This process continues until k seeds are selected.

### 2.5 Initial bicluster identification

Each seed initializes a bicluster. Let the current bicluster be


$$M_{R,C}={\left(M_{r,c}\right)}_{r\in R,c\in C},$$

where R and C denote the included row and column sets, respectively. A row u is randomly selected from this initial bicluster. For each external row x∉R, the LACS between row x and u is computed, and rows are ranked by descending LACS length. Rows are then iteratively added according to this ranking, while columns are continuously pruned to ensure that the column set  C remains the common subsequence shared by all rows in $A_{R\cdot }$, thereby maintaining the trend‑preserving property of $M_{R,C}$. Iteration stops when either of the following conditions is met:

1.
min⁡(|R|,|C|) decreases compared with the previous iteration.
2.
|C|<L·δ , where L is the LACS length of the initial seed pair and δ is a user-defined parameter (default value is 0.12).

### 2.6 Noise-aware row and column expansion

Noise-aware column expansion extends existing biclusters by selecting columns that satisfy admissible intervals and cross-row consensus. For each candidate column $c^{\prime }\notin C$, its potential insertion position is evaluated in every row r∈R. The admissible interval for row  r is defined as:


$$[L_{r}^{c^{\prime }},U_{r}^{c^{\prime }}]=[M_{r,c^{\prime }}\cdot (1-\gamma \cdot CV_{r}),M_{r,c^{\prime }}\cdot (1+\gamma \cdot CV_{r})],\;r\in R,$$

where $CV_{r}$ is the coefficient of variation of the row r across columns in C, and γ>0 is a user-defined scaling parameter (default value is  0.05) controlling noise tolerance. For each column c∈C, the matching score with candidate column $c^{\prime }$ is the number of rows whose entries fall within the admissible interval:


$$S\;(c,c^{\prime })=\left\{r:M_{r,c}\in [L_{r}^{c^{\prime }},U_{r}^{c^{\prime }}]\right\}.$$

Each candidate column is then evaluated by its maximum matching score against the existing bicluster columns. Specifically,


$$c^{*}=\arg\underset{c\in C}{\max}S(c,c^{\prime }),$$

where $c^{*}$ is the current bicluster column with the highest score and defines the candidate insertion position. Candidate column $c^{\prime }$ is accepted for insertion into the bicluster if


$$S(c^{*},c^{\prime })\geq \eta \cdot |R|,$$

where η is a user-defined parameter (default value is 0.8) controlling the required level of agreement across rows. This serves as the decision rule for determining whether the column satisfies the inclusion requirement. An illustrative comparison with strict column expansion is provided in Supplementary Fig. S3, available as supplementary data at *Bioinformatics* online.

After column expansion, rows not currently in R are examined. A row is added to the bicluster if its LACS length with row u has at least |C|·η.

### 2.7 Pruning of overlapping biclusters

After obtaining biclusters, a pruning step is performed to reduce pairwise overlaps. The area of bicluster $M_{R,C}$ is defined as |R|·|C|. For any two biclusters $M_{R_{1},C_{1}}$ and $M_{R_{2},C_{2}}$, if their overlap satisfies


$$|R_{1}\cap R_{2}|\cdot |C_{1}\cap C_{2}|\geq \epsilon \cdot \min(\left|R_{1}|\cdot |C_{1}|,|R_{2}|\cdot |C_{2}\right|),$$

where ϵ is a user-defined threshold (default value is 0.8), the bicluster with the smaller area is pruned by removing rows and columns from the overlapping submatrix, while its remaining rows and columns are retained. Thus, the overlap threshold controls the degree of overlap allowed in the final biclustering result.

### 2.8 Implementation details and parameter summary

NoiBic was implemented in C++17. Key parameters and their default values are listed in Supplementary Table S1, available as supplementary data at *Bioinformatics* online. Runtime benchmarking was performed on Intel Xeon Platinum 8260 CPUs at 2.40 GHz. Source code and usage instructions for NoiBic are publicly available at https://github.com/lcyi0208/NoiBic. The exact software version used in this study, v1.0.1, has been permanently archived on Zenodo at https://doi.org/10.5281/zenodo.22732377. Pseudocode is provided in Supplementary File 2. Software dependencies, runtime environment, benchmarking hardware, and resource allocation are detailed in Supplementary Sections 13-14, and Table S4, available as supplementary data at *Bioinformatics* online.

## 3 Experimental setup

### 3.1 Data overview

We evaluated NoiBic using both synthetic and real gene expression datasets. Synthetic datasets were generated for method comparison, ablation studies, and seed-selection analyses. Real-data evaluation included 17 datasets: six bulk transcriptomic datasets for functional gene module evaluation and 11 scRNA-seq datasets for benchmarking cell type-associated biclusters and conducting case studies. Synthetic settings are provided in Supplementary Section 5, and Supplementary Data 1, available as supplementary data at *Bioinformatics* online; real datasets are summarized in Supplementary Tables S2 and S3, available as supplementary data at *Bioinformatics* online.

### 3.2 Data preprocessing

Datasets were optionally preprocessed to ensure quality and comparability across benchmarks. The input expression matrix E, derived from microarray, bulk RNA-seq, or scRNA-seq data, contains genes as rows and samples or cells as columns. For microarray data with more than 30 000 genes, the top 20,000 genes ranked by coefficient of variation were retained. For scRNA-seq data, 2000 highly variable genes were selected, with optional dropout correction using MAGIC (van Dijk *et al.* 2018). The processed matrix was then row-wise min-max normalized to obtain M:


$$M_{i\cdot }=\frac{E_{i\cdot }-\min\left\{E_{i\cdot }\right\}}{\max\left\{E_{i\cdot }\right\}-\min\left\{E_{i\cdot }\right\}}.$$

### 3.3 Evaluation and benchmarking

We evaluated NoiBic and competing algorithms on simulated and real high-throughput transcriptomic datasets. Because real datasets lack ground-truth biclusters, simulated datasets with planted biclusters were used to evaluate agreement with known ground truth, using recovery, relevance scores (Prelić *et al.* 2006) and the clustering error similarity score (SCE) (Horta and Campello 2014). Notably, SCE is particularly useful for assessing overlapping biclustering structures. For real bulk datasets, detected gene modules were evaluated by KEGG pathway enrichment analysis, and the overall detection effectiveness was summarized by the F1 score (Liu *et al.* 2023). For scRNA-seq datasets, sample-level clustering quality was assessed using Adjusted Rand Index (ARI) (Hubert and Arabie 1985) and Normalized Mutual Information (NMI) (Strehl and Ghosh 2002), with known cell type labels as reference. Metric definitions and evaluation procedures are provided in Supplementary Section 11.

### 3.4 Benchmarking algorithms

We benchmarked NoiBic against classical biclustering methods FABIA (Hochreiter *et al.* 2010), ISA (Bergmann *et al.* 2003), OPSM (Ben-Dor *et al.* 2002), CPB (Bozdağ *et al.* 2009), QUBIC (Li *et al.* 2009), and UniBic (Wang *et al.* 2016), recent methods ARBic (Liu *et al.* 2023), RecBic (Liu *et al.* 2020), RUBIC (Sriwastava *et al.* 2023), and HBiC (José-García *et al*. 2024), and single-cell methods QUBIC2 (Xie *et al.* 2020) and scBC (Gong *et al.* 2024). scBC was evaluated only in the single-cell benchmark because it relies on scVI-based representation learning. Simulated datasets used default settings, with the reported bicluster number fixed when applicable; real-data analyses used shared settings and grid searches for selected method-specific parameters. Detailed settings are provided in Supplementary Section 8, and Supplementary Data 2, available as supplementary data at *Bioinformatics* online.

For single-cell benchmarking, we included methods applicable to scRNA-seq data or whose biclusters can be converted to non-overlapping cell partitions for ARI and NMI evaluation. QUBIC2, scBC, and NoiBic were directly applied to single-cell expression data, whereas overlapping biclusters from other methods were converted before scoring. Detailed conversion rules and exclusion criteria are provided in Supplementary Section 11, available as supplementary data at *Bioinformatics* online.

The implementation sources, software environments, benchmarking hardware, and resource allocations for the compared methods are summarized in Supplementary Sections 13-14, and Table S4, available as supplementary data at *Bioinformatics* online.

## 4 Results

### 4.1 Benchmarking on simulated and real transcriptomic datasets

#### 4.1.1 NoiBic accurately recovers biclusters from simulated noisy data

To evaluate agreement between identified and planted biclusters, we generated synthetic datasets using BiBench (Eren *et al.* 2013) and G-Bic (Castanho *et al.* 2023). Five factors were examined: bicluster coherency, matrix size, bicluster number, noise level, and overlap level. BiBench was used as the primary benchmark, while G-Bic introduced noise into both the background matrix and planted biclusters, providing a complementary and more complex noise setting.

On BiBench-generated datasets, NoiBic achieved the best overall performance across most simulation settings (Fig. 2). It showed strong results across bicluster coherency, matrix size, and bicluster number (Fig. 2A–I). Its advantages became more evident as noise and overlap levels increased, particularly for SCE under highly overlapping settings (Fig. 2O). These results indicate that NoiBic is particularly effective for noisy and overlapping biclustering structures.

**Figure 2:**
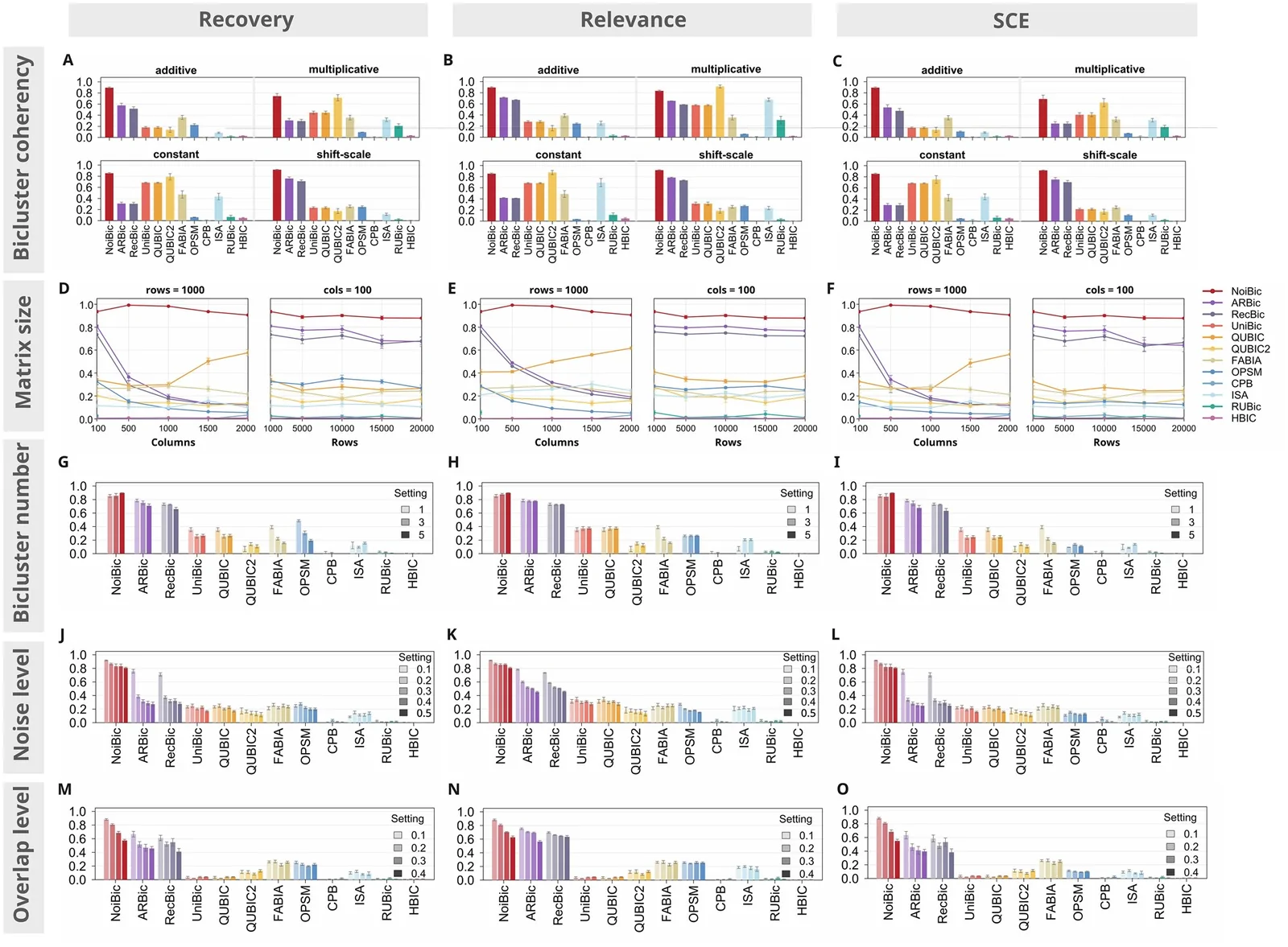
Performance of biclustering methods on BiBench simulations measured by recovery, relevance, and SCE. (A–C) Bicluster coherency. (D–F) Matrix size. (G–I) Bicluster number. (J–L) Noise level. (M–O) Overlap level.

G-Bic-generated datasets showed a similar pattern (Supplementary Fig. S4, available as supplementary data at *Bioinformatics* online). Although NoiBic was not the top method in a few local settings, such as low-noise datasets where RecBic performed well, its advantage increased with noise level and became strongest under higher-noise conditions (Supplementary Fig. S4J-L, available as supplementary data at *Bioinformatics* online).

#### 4.1.2 NoiBic remains robust under ablation and seed-selection analyses

We performed ablation and seed-selection analyses to assess key components of NoiBic. Replacing LACS-based seed identification with strict LCS matching or removing the noise-tolerant column-expansion criterion reduced performance, especially as noise increased (Supplementary Fig. S5A-C, available as supplementary data at *Bioinformatics* online). The default top-k seed strategy performed comparably to larger seed sets, whereas smaller seed sets reduced accuracy (Supplementary Fig. S5D-F, available as supplementary data at *Bioinformatics* online). Using all candidate seeds reduced accuracy under low-noise settings, suggesting that redundant seeds may introduce false-positive biclusters.

#### 4.1.3. NoiBic effectively identifies functional gene modules from noisy bulk transcriptomic data

For bulk transcriptomic datasets, gene modules were evaluated by KEGG enrichment and summarized using the F1 score. NoiBic performed strongly across the six datasets and outperformed all competing methods on HerBC, ecoli_dream5, and ecoli_colombos, which contained relatively larger numbers of samples (Fig. 3A). This suggests that NoiBic benefits from richer ordering information in high-throughput expression matrices while remaining competitive on smaller datasets.

**Figure 3:**
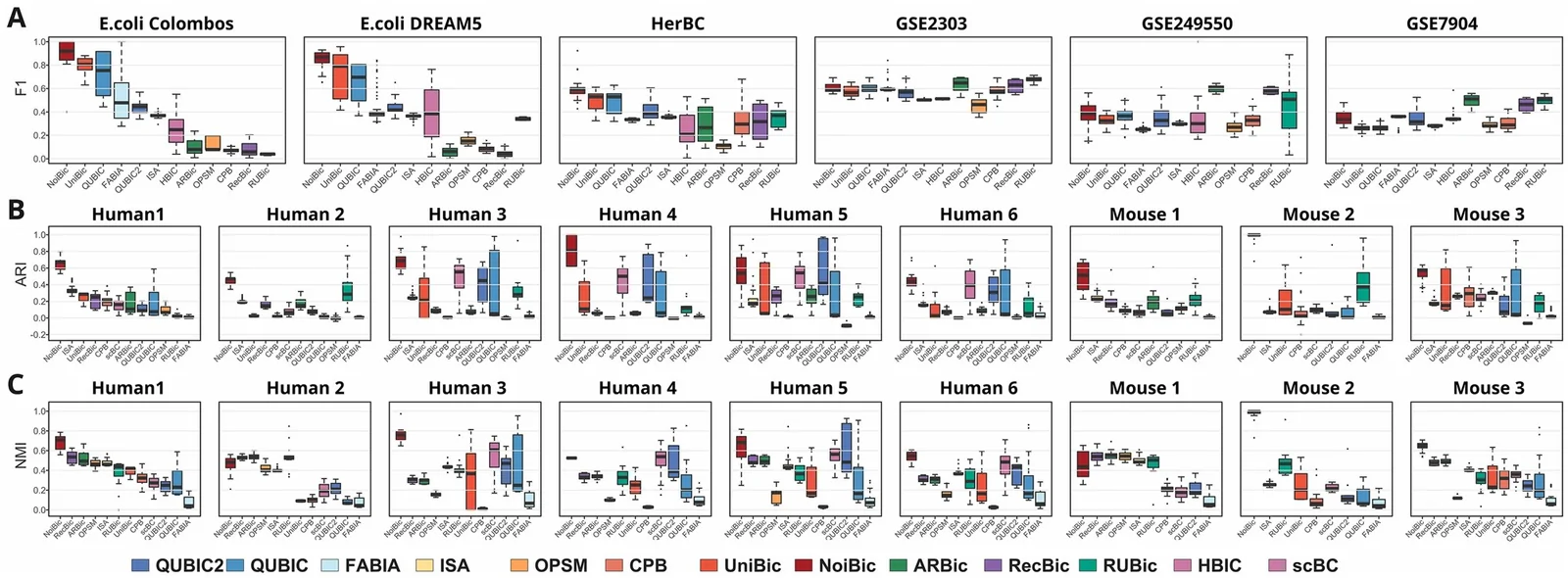
Performance comparison on real bulk and single-cell transcriptomic datasets. (A) KEGG enrichment-based F1 scores on six bulk datasets. (B, C) ARI and NMI scores, respectively, on nine annotated scRNA-seq datasets. Boxplots summarize performance across the tested parameter settings.

#### 4.1.4. NoiBic reliably recovers cell type-associated biclusters in noisy scRNA-seq datasets

We further evaluated cell type-associated biclusters on nine annotated scRNA-seq datasets using ARI and NMI. NoiBic generally outperformed competing methods (Fig. 3B and C), with significant overall advantages supported by Wilcoxon rank-sum tests after Benjamini-Hochberg correction (Supplementary Fig. S7, available as supplementary data at *Bioinformatics* online). Although scBC performed competitively on Human5, it used the known number of cell types as input. On Human1 and Mouse1, some methods achieved higher NMI by producing finer partitions, whereas NoiBic showed stronger ARI performance, indicating better agreement with known cell type labels.

#### 4.1.5. Runtime and memory usage of NoiBic

We further evaluated runtime and peak memory usage on representative BiBench simulated datasets with varying matrix sizes and on two real scRNA-seq datasets, Mouse2 and Mouse3. NoiBic showed favorable runtime and moderate memory usage, supporting its practical applicability to large-scale expression data (Supplementary Fig. S6; Supplementary Data 3, available as supplementary data at *Bioinformatics* online).

### 4.2 Case studies demonstrating biological interpretability

#### 4.2.1 NoiBic effectively identifies gene modules associated with pleomorphic adenoma

Pleomorphic adenoma is the most common benign salivary gland tumor, but its molecular basis remains unclear. We applied NoiBic to scRNA-seq data from human pleomorphic adenoma and selected the top six biclusters for downstream analysis. These biclusters showed distinct UMAP distributions and selective gene-expression patterns, indicating cell type specificity (Fig. 4A and B). Marker-gene analysis further showed that each bicluster contained genes associated with its corresponding cellular population (Supplementary Fig. S8, available as supplementary data at *Bioinformatics* online).

**Figure 4:**
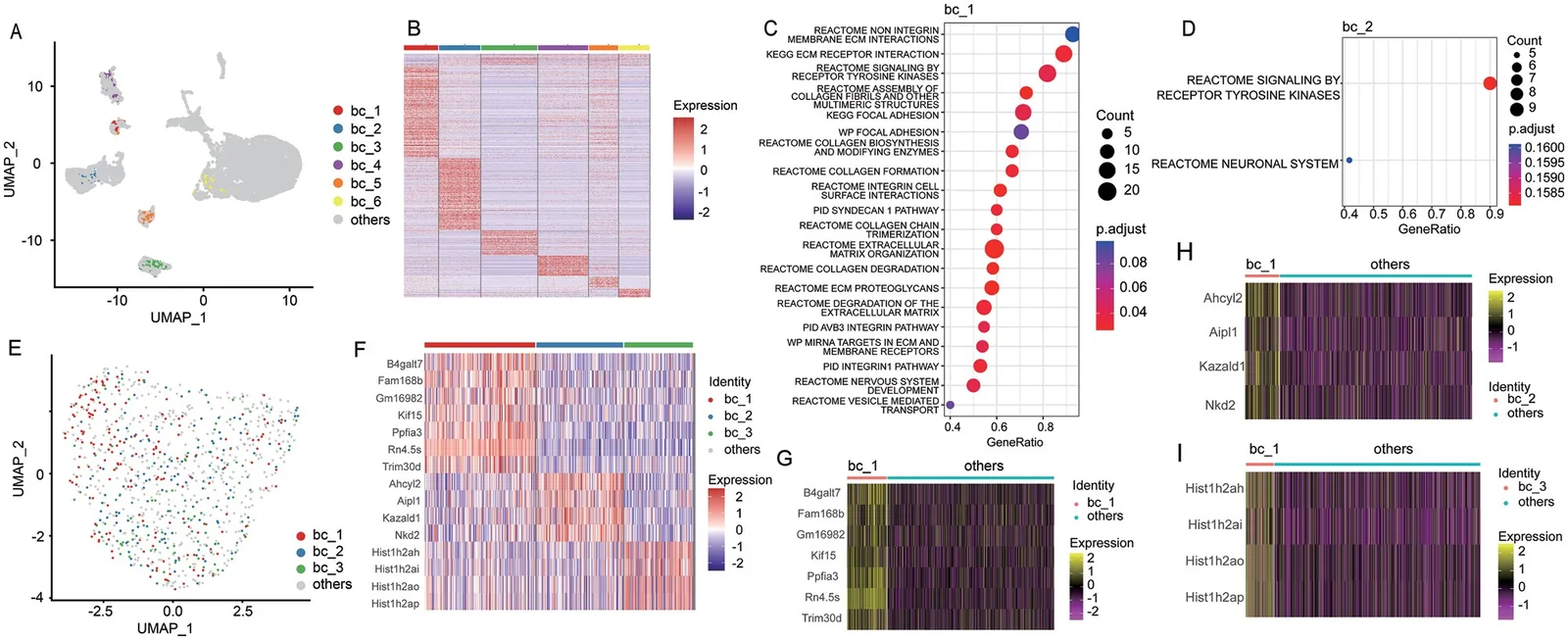
Biological interpretation of NoiBic-identified gene modules in two scRNA-seq case studies. (A–D) Analysis of human pleomorphic adenoma data. (A) UMAP of six NoiBic biclusters. (B) Gene-expression heatmap across biclusters. (C, D) GSEA results for biclusters 1 and 2. (E–I) Analysis of mouse embryonic stem cell data. (E) PCA-UMAP colored by NoiBic bicluster assignments. (F) Representative-gene heatmap across biclusters. (G–I) Heatmaps comparing representative-gene expression between bicluster cells and remaining cells.

Pathway enrichment analysis revealed biological programs relevant to pleomorphic adenoma (Subramanian *et al.* 2005). Genes in bicluster 1 were enriched in collagen biosynthesis and extracellular matrix-related pathways, suggesting altered tumor-ECM interactions (Fig. 4C). Bicluster 2 was enriched in receptor tyrosine kinase signaling, which regulates cell growth, survival, and differentiation (Fig. 4D) (Huang *et al.* 2021).

Together, these results show that NoiBic identifies pleomorphic adenoma-associated cell clusters and gene modules linked to disease-relevant pathways.

#### 4.2.2 NoiBic identifies gene modules associated with distinct states in undifferentiated mouse embryonic stem cells

We next applied NoiBic to 935 undifferentiated mouse embryonic stem cells. Conventional PCA-UMAP visualization showed limited separation, whereas NoiBic identified three biclusters representing gene modules associated with distinct cellular states (Fig. 4E and F). Heatmaps of representative genes and their corresponding cell subsets showed distinct expression patterns across the three biclusters (Fig. 4F–I).

Analysis using Seurat (Stuart *et al.* 2019) showed elevated scores for each module in its corresponding bicluster (Supplementary Fig. S9A-F, available as supplementary data at *Bioinformatics* online). Marker-gene analyses further showed that genes in the three modules had positive log2 fold-changes and significant adjusted *P*-values (Supplementary Fig. S9G-I, available as supplementary data at *Bioinformatics* online).

Several genes in bicluster 3, including Hist1h2ah, Hist1h2ai, Hist1h2ao, and Hist1h2ap, belong to the histone H2A family (Fig. 4I; Supplementary Fig. S9I, available as supplementary data at *Bioinformatics* online). Because histones regulate chromatin architecture and embryonic development (Wu *et al.* 2014), their coordinated expression suggests a potential role in mESC cell state heterogeneity.

## 5 Discussion

In this study, we systematically evaluated NoiBic for identifying context-associated gene modules from noisy transcriptomic data. Across BiBench and G-Bic simulations, NoiBic showed robust bicluster identification across varying bicluster coherency, matrix size, bicluster number, noise level, and overlap level settings, with the clearest gains under high-noise and overlapping conditions. Ablation analyses supported the contributions of LACS-based seed identification and noise-aware column expansion, while the default top-k strategy balanced accuracy and computational efficiency. On real datasets, NoiBic identified functionally enriched modules in bulk transcriptomic data and cell type-associated biclusters in scRNA-seq data, including modules associated with disease and cellular states in the two case studies.

These results highlight the main strengths of NoiBic relative to existing biclustering methods: noise-tolerant seed identification and noise-aware column expansion. Co-expression network approaches are valuable for unsupervised gene-module discovery based on gene-gene relationships, but they primarily characterize global co-expression patterns and do not directly identify the specific subsets of samples or cells where each module is active. In contrast, NoiBic jointly identifies gene sets and their associated sample or cell subsets, enabling the discovery of context-associated modules from noisy expression matrices. Although this study focused on transcriptomic data, NoiBic operates on numerical matrices and may therefore be applicable to other data types after appropriate preprocessing.

NoiBic still has limitations. It is designed to identify trend-preserving modules with overlap controlled by a user-defined threshold. Distinct non-overlapping or partially overlapping biclusters can be retained, whereas highly redundant or nested structures with nearly identical row or column supports may be merged or pruned. Optional MAGIC-based dropout correction can increase computational cost for large scRNA-seq datasets. Overall, NoiBic provides a robust and interpretable framework for functional gene module discovery in high-throughput transcriptomic data.

## Supplementary Material

btag743_Supplementary_Data
