# Supplementary material for: NoiBic: a noise-tolerant biclustering algorithm for high-throughput gene expression data analysis
Source: Bioinformatics. 2026 Oct 7;42(10):btag743. doi: 10.1093/bioinformatics/btag743 (PMC13681661; doi:10.1093/bioinformatics/btag743)
Supplement: Supplementary file 1 [file btag743_supplementary_data.zip › Supplementary File 1.pdf]

Supplementary materials for the manuscript

1. Schematic examples of bicluster patterns

Several bicluster pattern terms are used in the manuscript and in the synthetic dataset experiments, including constant, shift, scale, shift-scale, order-preserving, and trend-preserving patterns. To help readers interpret these terms, we provide schematic examples of the major pattern classes in Fig. S1. The examples illustrate how rows within a bicluster may share identical values, additive offsets, multiplicative factors, combined shift-scale effects, consistent condition ordering, or similar trend shapes. The noise-tolerant trend-preserving example further illustrates the approximate pattern structure targeted by NoiBic under noisy expression measurements.

a. Constant

	c1	c2	c3
g1	1	1	1
g2	1	1	1
g3	1	1	1

same value

b. Shift

	c1	c2	c3
g1	1	2	3
g2	2	3	4
g3	3	4	5

additive offsets

c. Scale

	c1	c2	c3
g1	1	2	3
g2	2	4	6
g3	3	6	9

multiplicative factors

d. Shift-scale

	c1	c2	c3
g1	2	3	4
g2	3	5	7
g3	4	7	10

combined offsets and factors

e. Order-preserving

	c1	c2	c3
g1	1	7	5
g2	2	8	6
g3	-1	4	2

same order: c1 < c3 < c2

f. Trend-preserving

	c1	c2	c3
g1+	1	7	5
g2+	2	8	6
g3-	-1	-7	-5

order: c1 < c3 < c2
g3 follows reverse order

g. Noise-tolerant trend-preserving

	c1	c2	c3
g1+	1	4.9	5
g2+	2	8	6
g3-	-1	-7	-5

order: c1 < c3 < c2
g3 follows reverse order under noise

Fig. S1. Schematic examples of major bicluster patterns. Representative examples are shown for constant, shift, scale, shift-scale, order-preserving, trend-preserving, and noise-tolerant trend-preserving bicluster patterns. The schematic helps clarify pattern terminology used in the manuscript and synthetic dataset experiments.

2. A simple example demonstrating the advantage of LACS over LCS

Given two sequences $M_1 = \{0, 0.2, 0.4, 0.41, 0.8, 1\}$ and $M_2 = \{0.8, 0, 0.21, 0.2, 1, 0.6\}$, we compare the results obtained by the LCS and LACS algorithms. The length of the longest common subsequence (LCS) is 3, whereas LACS yields a subsequence of length 4. Under the LACS framework, elements $M'_{1,3} = 0.4$ and $M'_{1,4} = 0.41$ are regarded as exchangeable, since the exchanged values and their corresponding original values are sufficiently similar; that is, any pairwise combination among these four values satisfies the CV-scaled proximity condition:

$$\frac{|m_p - m_q|}{\min(|m_p|, |m_q|)} \leq CV_1 \cdot \alpha,$$

where $CV_1 = 0.73$ and $\alpha = 0.2$. Similarly, $M'_{2,2} = 0.2$ and $M'_{2,3} = 0.21$ also satisfy this condition and are treated as exchangeable. For each sequence, we compute the exchange cost:

$$score_1 = \frac{fabs(\mathrm{M}'_{1,3} - M'_{1,4})}{\min(fabs(\mathrm{M}'_{1,3}), fabs(M'_{1,4})) * CV_1} = 0.034,$$

$$score_2 = \frac{fabs(\mathrm{M}'_{2,2} - M'_{2,3})}{\min(fabs(\mathrm{M}'_{2,2}), fabs(M'_{2,3})) * CV_2} = 0.065.$$

Since $score_2 > score_1$, the exchange in M_1 is preferred. As a result, LACS allows a local reordering between $M'_{1,3}$ and $M'_{1,4}$, which increases the matching score. In particular, the dynamic programming entry $C_{3,2}$ is updated to 2 under LACS, compared to 1 under the standard LCS algorithm. Therefore, by permitting local exchanges between nearby elements within a controlled range, LACS effectively accounts for noise-induced fluctuations and enables a more tolerant identification of common subsequences between two sequences.

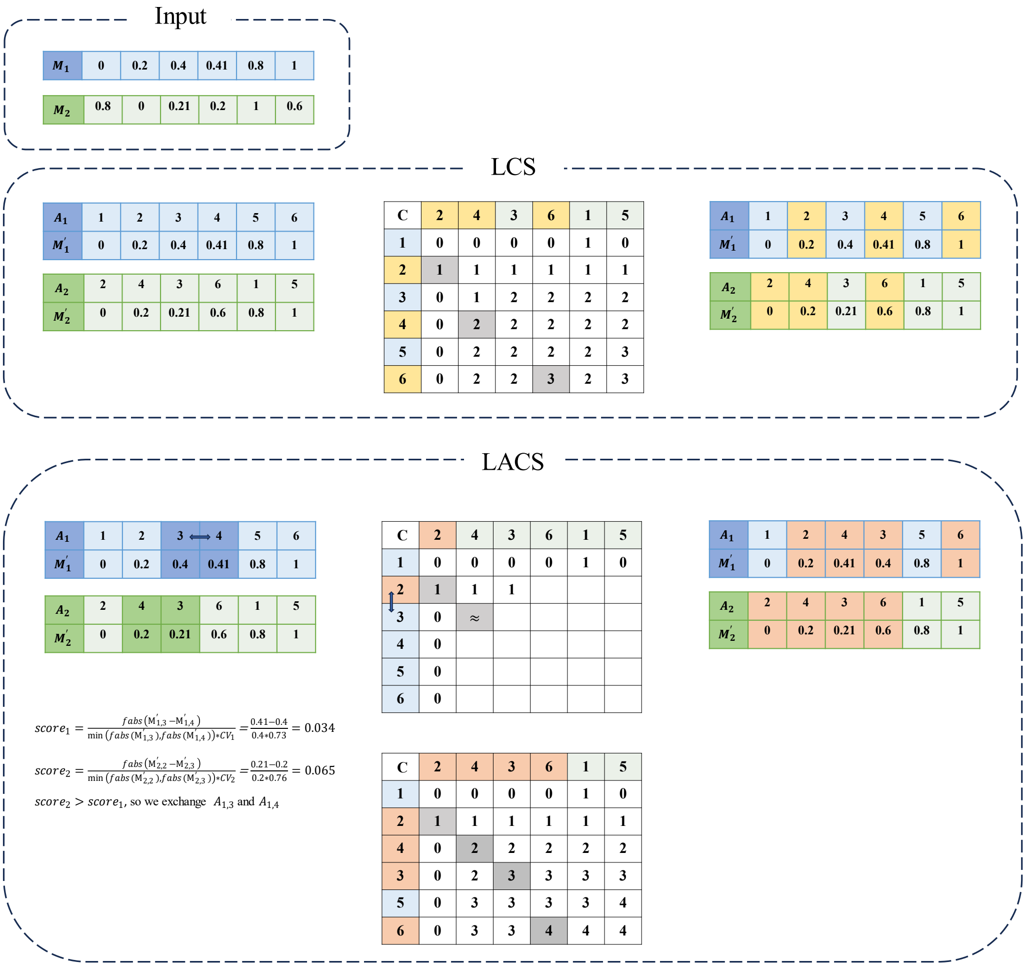

Fig. S2. Example demonstrating how LACS accounts for noise-induced fluctuations. Through controlled local exchanges between nearby elements, LACS enables more flexible subsequence matching compared to classical LCS.

3. A simple example demonstrating the advantage of noise-aware column expansion over baseline expansion

The upper part of Fig. S3 illustrates the case in which the baseline column expansion procedure is applied. In this setting, the yellow marks indicate the feasible insertion positions of each element in the candidate column. Under the strict expansion criterion, however, no common column position can be identified that allows the entire candidate column to be inserted into the current bicluster. In contrast, the lower part of Fig. S3 shows the result of applying the noise-aware column expansion. The matrix to the right of the candidate column records, for each row, the variables required to compute the insertable interval, including the row-specific CV value and the corresponding interval endpoints val_l and val_r. To account for noise-induced fluctuations, the insertable interval of each row is relaxed within an allowable tolerance range, which increases the number of feasible insertion positions for each element, as highlighted in orange. As a result, the candidate column becomes insertable, and its final insertion position is indicated by the blue box in Fig. S3.

Baseline column expansion

	c_1	c_2	c_3	c_4	c_5	c_6	c_7	c_8	c_9	c
r_1	1	3	5	8	9	9	13	15	17	9
r_2	1	3	4.9	5	5.5	11	13	15	17	5
r_3	1	3	4	4.5	5.1	5.2	13	15	17	5
r_4	1	3	5	7	10	11	11.5	15	17	11
r_5	-1	-2.5	-3.1	-3.1	-3.3	-11	-13	-15	-17	-3
r_6	-1	-3	-5	-8	-9	-10	-13	-15	-17	-9

Noise-aware column expansion via insertable intervals

	c_1	c_2	c_3	c_4	c_5	c_6	c_7	c_8	c_9	c	CV	val_l	val_r
r_1	1	3	5	8	9	9	13	15	17	9	0.5723	7.969903	10.0301
r_2	1	3	4.9	5	5.5	11	13	15	17	5	0.6435	4.3565	5.6435
r_3	1	3	4	4.5	5.1	5.2	13	15	17	5	0.7291	4.270864	5.729136
r_4	1	3	5	7	10	11	11.5	15	17	11	0.5667	9.753297	12.2467
r_5	-1	-2.5	-3.1	-3.1	-3.3	-11	-13	-15	-17	-3	-0.768	-2.53895	-3.46105
r_6	-1	-3	-5	-8	-9	-10	-13	-15	-17	-9	-0.567	-7.9802	-10.0198

Fig. S3. Example comparing baseline and noise-aware column expansion. Baseline expansion fails to identify a common insertion position, whereas noise-aware expansion enables successful insertion of the candidate column by relaxing insertable intervals within an allowable tolerance range.

4. Summary of NoiBic Parameters

To improve clarity and facilitate practical use of the NoiBic framework, we summarize the key algorithmic parameters introduced in the Methods section in Table S1. The table lists the parameters as defined in the manuscript, their corresponding names used in the software implementation, along with brief descriptions and default values. These parameters correspond to different stages of the NoiBic algorithm, including LACS-based seed identification, bicluster expansion, and overlap pruning. While Table S1 focuses on the core algorithmic parameters described in the manuscript, additional implementation-related options and runtime configurations are available in the software package. Detailed usage can be found in the GitHub repository or accessed via command-line help (e.g., using "--help" or "-h").

Table S1. Summary of key parameters used in the NoiBic framework. The table lists the parameters defined in the Methods section, their corresponding implementation names, descriptions, and default values.

Parameter (Manuscript)	Parameter (Implementation)	Description	Default
α	l	Permissible mismatch rate during element swapping when searching for the Longest Common Subsequence (LCS) between two sequences. Floating-point value in the range [0, 1]	0.1
η	e	Permissible error rate during bicluster expansion. Floating-point value in the range (0.5, 1]	0.8
ϵ	f	Overlap filtering threshold for biclusters. Floating-point value in the range [0, 1]	0.8
δ	n	Minimum cluster width as a fraction of the original seed length. Floating-point value in the range [0, 1]	0.12
γ	b	Permissible numerical error for column expansion using binary search. Floating-point value in the range [0, 1], or 2 to disable column expansion.	0.05
k	k	Seed number multiplier. seed_num is derived as k * o.	20
o	o	Number of biclusters to report	30

5. Detailed information about synthetic dataset.

Three groups of synthetic datasets were generated for different experimental purposes. The first two groups were generated using G-Bic and BiBench, respectively, and were used to compare the performance of NoiBic with other biclustering methods under different simulation settings. The third group was generated using BiBench and was used for ablation studies and seed-selection analyses. Detailed parameter settings for all synthetic dataset generation experiments are provided in Supplementary Data 1.

Datasets for method comparison. For the G-Bic- and BiBench-based method-comparison datasets, we evaluated five simulation factors: bicluster coherency, number of biclusters, noise level, overlap level, and matrix size. For the G-Bic-based datasets, the bicluster coherency included additive, constant, multiplicative, and order-preserving patterns. For the BiBench-based datasets, the bicluster coherency included additive, constant, multiplicative, and shift-scale patterns. The number of planted biclusters was set to 1, 3, or 5; the noise level was set to 0.1, 0.2, 0.3, 0.4, or 0.5; and the overlap level was set to 0.1, 0.2, 0.3, or 0.4. Matrix size was evaluated by varying rows (1,000, 5,000, 10,000, 15,000, and 20,000) with columns fixed at 100, and by varying columns (100, 500, 1,000, 1,500, and 2,000) with rows fixed at 1,000.

For these method-comparison datasets, 10 replicates were generated for each parameter setting. Unless otherwise evaluated, the default matrix size was 10,000 rows by 100 columns, the default noise level was 0.3, the planted biclusters were non-overlapping, and three biclusters were planted. The default bicluster coherency was order-preserving for the G-Bic-based datasets and shift-scale for the BiBench-based datasets. The default bicluster size was 1,000 rows by 20 columns. In the matrix-size experiments, the bicluster size was set to approximately 10% of the number of rows and columns, with a minimum size constraint to avoid excessively small biclusters.

For the G-Bic-based group, numeric real-valued matrices were generated with values restricted to [-20, 20] and a background distribution based on $N(0,1)$. For order-preserving biclusters, the row pattern was set to 'None', the column pattern was set to 'OrderPreserving', and the time profile was set to 'MonotonicallyIncreasing'. In overlap experiments, the settings were plaid_coherency = None, overlapping_biclusters = 100%, and overlapping_rows and overlapping_columns equal to the specified overlap level. Under plaid_coherency = None, overlapping entries were not combined additively, multiplicatively, or by interpolation; instead, the later generated bicluster value was retained. For G-Bic noise settings, noise was applied to both the background and planted biclusters, with the default configuration noise_background = 30%, noise_biclusters = 30%, and noise_deviation = 1.

For the BiBench-based group, background entries were sampled from $N(0,1)$ and clipped to [-20, 20]. Constant, additive, and multiplicative biclusters were generated using the built-in BiBench functions. The shift-scale setting was generated by first creating an increasing

base vector, x_{base}, with length equal to the number of bicluster columns. For each row i, a shift parameter a_i and a positive scale parameter b_i were randomly generated, and the entries were computed as:

$$x_{ij} = a_i + b_i \cdot x_{base\,j}.$$

Because b_i was positive, rows could have different shifts and amplitudes while preserving the same column ordering within each bicluster. Because the data were generated by this shift-scale formulation, we refer to this BiBench-based setting as shift-scale in the main text, Fig. 2, and Supplementary Data 1. In the BiBench-based noise experiments, noise was added only to entries inside the planted biclusters: when the noise level was p, a proportion p of bicluster entries was perturbed by additive noise sampled from $N(0, 1)$. In overlap experiments, adjacent biclusters shared the specified proportion of rows and columns, and overlapping entries retained the value from the later planted bicluster.

Additional datasets for ablation and seed-selection analyses. For ablation studies and seed-selection analyses, we generated an additional group of BiBench-based datasets. Each dataset contained 1,000 rows and 500 columns, with three planted shift-scale biclusters and no overlap. Each bicluster covered 10% of the matrix size, corresponding to 100 rows and 50 columns. Four noise levels were considered: 0, 0.1, 0.2, and 0.3. For each noise level, 20 replicate datasets were generated.

6. Detailed information about the real bulk datasets.

To assess the performance of the compared biclustering methods on real bulk transcriptomic data, we collected six publicly available datasets, as analyzed in Section 4.1.3. These datasets span both microarray and RNA-seq platforms and cover multiple species, including Escherichia coli, Rattus norvegicus, and Homo sapiens. They also vary substantially in dimensionality, with a broad range of genes and experimental conditions, thereby providing a diverse benchmark for evaluating method performance across different biological settings and data scales.

Table S2. Summary of bulk transcriptomic datasets used for benchmarking. For each dataset, the table reports the number of genes, number of conditions, species, data type, and accession information.

Datasets	Number of genes	Number of conditions	Species	Data type	Accession
E. coli Colombos	2,093	2,470	Escherichia coli	Microarray data	doi.org/10.5281/zenodo.1157938
E. coli DREAM5	2,442	805	Escherichia coli	Microarray data	doi.org/10.5281/zenodo.1157938
GSE7904	54,675	62	Homo sapiens	Microarray data	GSE7904
GSE2303	8,799	94	Rattus norvegicus	Microarray data	GSE2303
GSE249550	23,091	93	Homo sapiens	Microarray data	GSE249550
HerBC	16,261	151	Homo sapiens	RNA-seq	http://lifeome.net/database/bcma/

7. Detailed information about the real single cell datasets.

We collected eleven publicly available single-cell RNA-seq datasets to evaluate the performance of the proposed method and to conduct downstream case studies. The first nine datasets were used for benchmarking, while the remaining two datasets were used for downstream case analyses. These datasets span both human and mouse samples and vary substantially in terms of gene number, variable gene selection, and cell counts after filtering, providing a diverse setting for evaluating method performance and biological interpretability.

Table S3. Summary of single-cell RNA-seq datasets used for benchmarking and case studies. The table reports, for each dataset, the number of genes, number of variable genes, number of cells after filtering, species, data type, and accession information.

Datasets	Number of genes	Number of variable genes	Number of cells after filtering	Species	Data type	Accession
Human1	18,539	3,273	1,715	Homo sapiens	single-cell RNA-seq	GSE76381
Human2	19,528	4,092	1,977	Homo sapiens	single-cell RNA-seq	GSE76381
Human3	11,786	1,916	860	Homo sapiens	single-cell RNA-seq	GSE126906
Human4	20,125	2,176	1,724	Homo sapiens	single-cell RNA-seq	GSE84133
Human5	20,125	1,718	3,605	Homo sapiens	single-cell RNA-seq	GSE84133
Human6	16,381	2,069	1,303	Homo sapiens	single-cell RNA-seq	GSE84133
Mouse1	19,259	4,166	1,907	Mus musculus	single-cell RNA-seq	GSE76381
Mouse2	16,349	2,389	12,648	Mus musculus	single-cell RNA-seq	GSE149751
Mouse3	19,972	4,040	3,005	Mus musculus	single-cell RNA-seq	GSE60361
Human pleomorphic adenoma	36,601	3,952	4,562	Homo sapiens	single-cell RNA-seq	GSE294254
Mouse embryonic stem cells	24,174	5,210	935	Mus musculus	single-cell RNA-seq	GSE65525

8. The parameters used by algorithms

Parameter settings were adjusted according to the dataset type. For simulated datasets, all applicable methods were run using their default parameters, except that the number of reported biclusters was fixed to the number of planted biclusters when applicable. For bulk transcriptomic datasets, the number of reported biclusters was fixed at 100 and all columns were retained as input; selected method-specific parameters were evaluated over predefined grid-search ranges, with default values included in the search range. For single-cell RNA-seq datasets, methods were included if they could be applied to scRNA-seq data or converted into non-overlapping cell partitions for ARI and NMI evaluation. Detailed parameter settings, including default values and search ranges, are summarized in Supplementary Data 2.

9. Performance evaluation on G-Bic simulated datasets

To further evaluate the robustness of different biclustering algorithms, we conducted additional simulations using the G-Bic framework. These datasets cover multiple challenging settings, including varying bicluster numbers, overlap levels, noise levels, bicluster coherency settings, and bicluster sizes. The performance of each method was assessed using recovery, relevance, and SCE scores. This supplementary analysis provides an additional benchmark to examine whether the observed performance trends are consistent across an independent simulation framework.

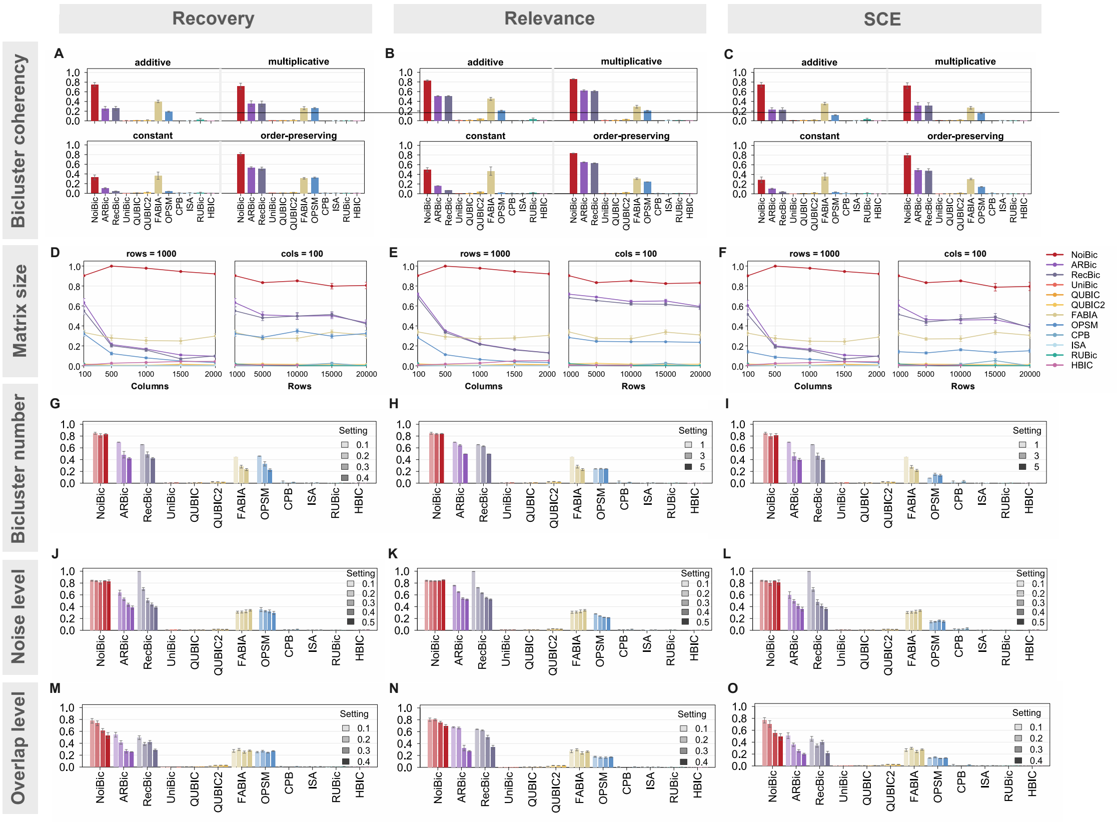

Fig. S4. Performance comparison on G-Bic simulated datasets. Performance comparison of biclustering algorithms on simulated datasets generated using G-Bic, measured by recovery, relevance, and SCE scores. (A-C) Bicluster coherency. (D-F) Matrix size. (G-I) Bicluster number. (J-L) Noise level. (M-O) Overlap level.

10. Ablation and seed-selection analyses

Ablation and seed-selection analyses were performed on BiBench-generated simulated datasets used for the ablation experiments. Each dataset contained 1,000 rows and 500 columns, with three planted shift-scale biclusters and no overlap. Each bicluster covered 100 rows and 50 columns. Four noise levels were evaluated: 0, 0.1, 0.2, and 0.3. For each noise level, 20 replicate datasets were generated.

For the ablation analysis, the full NoiBic model was compared with two ablated variants. In the strict LCS matching variant, the LACS-based seed identification step was replaced by strict longest common subsequence matching. In the strict column expansion variant, the noise-tolerant criterion used during column expansion was removed.

For the seed-selection analysis, we evaluated different values of k for selecting top-ranked candidate seeds. The default setting was $k = 20o$, where o is a user-defined parameter. Alternative settings included $k = o$, $5o$, and $100o$, as well as using all candidate seeds. Recovery, relevance, and SCE were used as evaluation metrics.

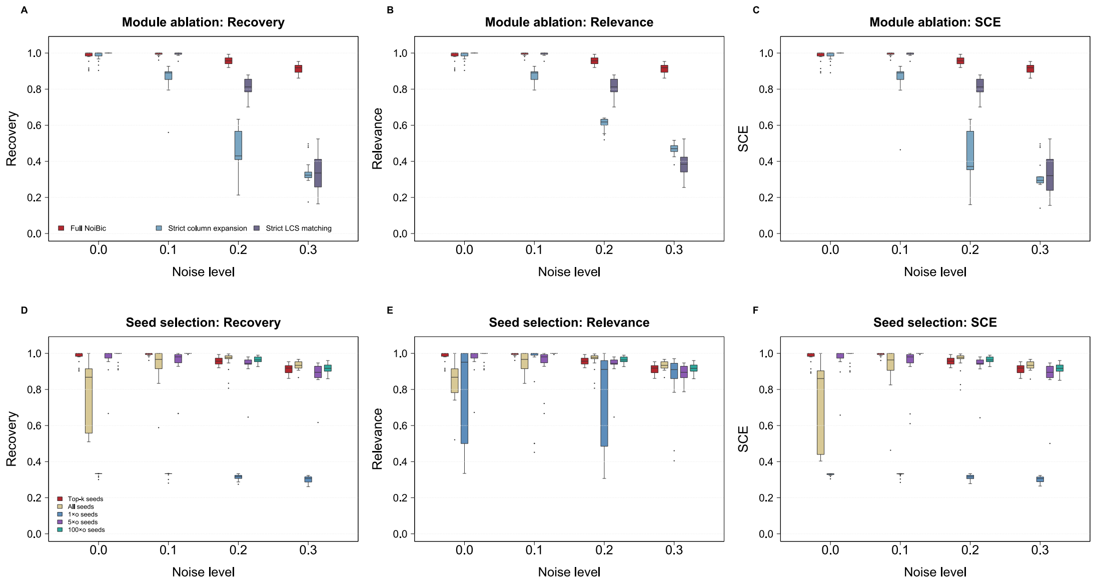

Fig. S5. Ablation and seed-selection analyses of NoiBic on simulated datasets. (A-C) Recovery, relevance, and SCE scores of the full NoiBic model and two ablated variants across different noise levels. Strict LCS matching replaces LACS-based seed identification with strict longest common subsequence matching, while strict column expansion removes the noise-tolerant criterion during column expansion. (D-F) Recovery, relevance, and SCE scores under different seed-selection strategies. The default top-k strategy uses $k = 20o$, where o is a user-defined parameter. Alternative strategies include $k = o$, $5o$, and $100o$, as well as using all candidate seeds. Higher scores indicate better agreement with the planted biclusters.

11. Evaluation metrics

Metrics on simulated datasets. For simulated datasets, the embedded biclusters were used as ground truth. Let B_1 and B_2 denote the true and predicted sets of biclusters, respectively. For a bicluster, its row and column jointly define the matrix entries covered by that bicluster. We first evaluated performance using the match score introduced by Prelic et al. The match score between two biclustering results was defined as:

$$S(B_1, B_2) = \frac{1}{|B_1|} \sum_{b_1 \in B_1} \max_{b_2 \in B_2} J(b_1, b_2),$$

where the pairwise similarity between two biclusters was computed by the Jaccard coefficient:

$$J(b_1, b_2) = \frac{|b_1 \cap b_2|}{|b_1 \cup b_2|}.$$

Let B_{true} denote the set of embedded biclusters and B_{pred} denote the set of biclusters predicted by a method. The recovery and relevance scores were calculated by reversing the order of the two inputs to the match score:

$$Recovery = S(B_{true}, B_{pred}),$$
$$Relevance = S(\ B_{pred}, B_{true}).$$

Recovery measures how well the embedded biclusters were recovered, whereas relevance measures how well the predicted biclusters corresponded to the embedded biclusters. Higher values indicate better agreement.

In addition, we calculated the clustering-error-based similarity score, denoted as SCE, following the formulation reviewed by Horta and Campello. This measure compares the covered matrix entries of two biclustering results while accounting for overlapping biclusters. Let N_{rc} and $N_{rc}^{'}$ be the numbers of biclusters covering matrix entry (r, c) in B_{pred} and B_{true}, respectively. The overlap-aware union size is defined as:

$$|U| = \Sigma_{(r,c)} \max\{N_{rc}, N_{rc}^{'}\}.$$

Let $B_{pred} = \{B_{pred}^i\}_{i=1}^k$ denote the predicted biclustering result and $B_{true} = \{B_{true}^j\}_{j=1}^q$ denote the true biclustering result. Each predicted bicluster B_{pred}^i is represented by its row set R_{pred}^i and column set C_{pred}^i, and thus covers the matrix-entry set $R_{pred}^i \times C_{pred}^i$. The true biclusters are represented analogously by R_{true}^j and C_{true}^j. Let M be a one-to-one matching between biclusters in B_{pred} and B_{true}. The matching M contains $\min\{k, q\}$ matched pairs. For each matched pair, the overlap is computed as the number of shared matrix entries. The maximum matched overlap is:

$$d_{max} = \max_{M} \sum_{(i,j)\in M} \left|\left(R_{pred}^{i} \times C_{pred}^{i}\right) \cap \left(R_{true}^{j} \times C_{true}^{j}\right)\right|.$$

The SCE score was then calculated as:

$$SCE\left(B_{pred}, B_{true}\right) = \frac{d_{max}}{|U|}.$$

SCE ranges from 0 to 1, with larger values indicating greater agreement between the predicted and true biclustering results.

Metrics on real datasets. For real datasets, ground-truth biclusters were unavailable; therefore, evaluation was based on biological interpretability and sample-label agreement. For bulk microarray and RNA-seq datasets, gene modules identified by each method were evaluated using pathway enrichment analysis. Gene modules within each bicluster were evaluated using the F_1 score. A bicluster is considered enriched if it is significantly enriched for at least one KEGG pathway (adjusted p-value < 0.05) based on the Benjamini–Hochberg procedure, as implemented in the R package clusterProfiler. If an algorithm produces k biclusters and l of them are enriched, the F-score is computed as follows:

$$F = \frac{l}{k}.$$

For a bicluster $B = (I, J)$, where I is the gene set and J is the sample set, the gene enrichment score g is computed as

$$g = \frac{\left|\left(\sum_{i=1}^{p} K_i\right) \cap I\right|}{|I|},$$

where K_i $(i = 1,2,\cdots,p)$ are the pathways enriched by B. The overall G-score is then calculated as

$$G = \frac{1}{k}\sum_{i=1}^{k} g_i.$$

Finally, the F_1 score used to evaluate an algorithm's ability to detect gene modules is defined as

$$F_1 \text{ score} = \frac{2(F \times G)}{F + G}.$$

For single-cell RNA-seq datasets, known cell type labels were used as reference labels. The predicted cell groupings were compared with the known labels using Adjusted Rand Index (ARI) and Normalized Mutual Information (NMI). Higher ARI and NMI values indicate better agreement with the annotated cell types.

Conversion of biclustering results for single-cell evaluation. For single-cell datasets, ARI and NMI require a non-overlapping cell partition. Therefore, biclustering outputs were converted into cell labels before scoring. For methods that supported non-overlapping bicluster output, the corresponding overlap-control parameter was set to disable or restrict overlap. These methods included NoiBic, QUBIC, UniBic, QUBIC2, ARBic, and RecBic. For methods that did not support non-overlapping bicluster output, overlapping cell assignments were removed before computing ARI and NMI. These methods included FABIA, ISA, OPSM, CPB, and RUBIC. ARBic and RecBic returned only one bicluster on these datasets and were therefore not included in the final single-cell comparison. HBiC was excluded because it did not complete within the predefined runtime limit on large-scale scRNA-seq datasets. scBC was evaluated only on single-cell datasets because it relies on scVI-based representation learning; its cluster number was set to the known number of cell types, as required by the method.

12. Runtime and memory usage comparison

Runtime and peak memory usage were evaluated on representative simulated and real datasets. For simulated data, one BiBench-generated dataset was selected from each matrix-size setting, including settings with fixed columns and varying rows, and settings with fixed rows and varying columns. To assess practical scalability on real single-cell data, runtime and memory usage were also measured on Mouse2 and Mouse3. RecBic and ARBic did not complete on Mouse2 because of the large data scale, and HBiC was not included in the real single-cell benchmark because it did not complete within the predefined runtime limit. Detailed runtime and peak memory measurements are provided in Supplementary Data 3.

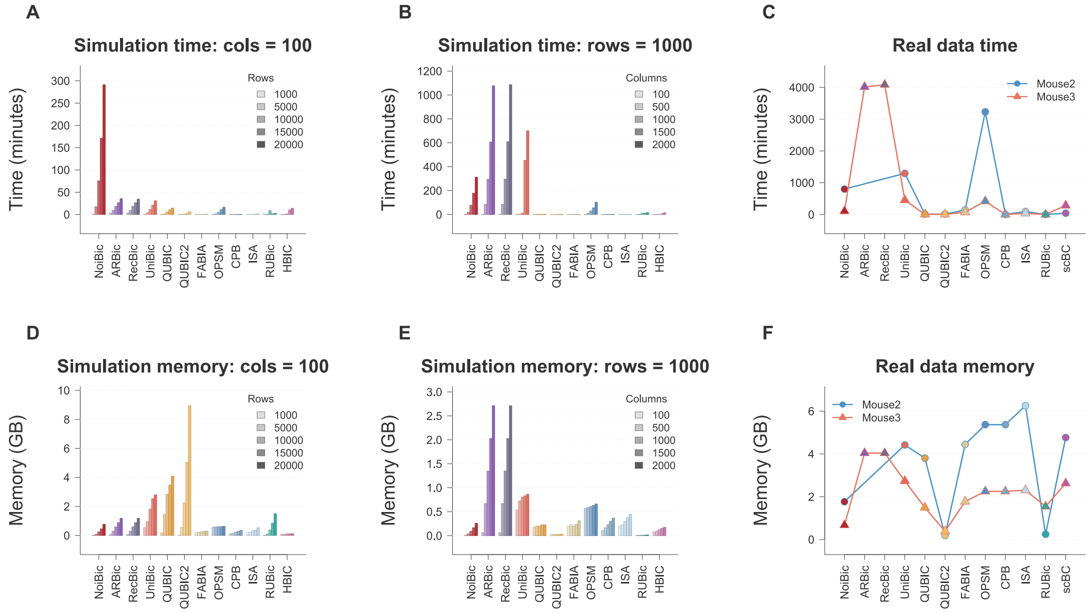

Fig. S6. Runtime and memory usage comparison of biclustering methods. (A-B) Runtime on representative BiBench-generated simulated datasets with varying matrix sizes. (A) Columns were fixed at 100 and rows were varied. (B) Rows were fixed at 1,000 and columns were varied. (C) Runtime on two real scRNA-seq datasets, Mouse2 and Mouse3. (D-E) Peak memory usage on the corresponding simulated datasets. (F) Peak memory usage on Mouse2 and Mouse3. Missing values indicate methods that did not complete or were not included under the corresponding benchmark setting. RecBic and ARBic did not complete on Mouse2 because of the large data scale, and HBiC was not included in the real single-cell benchmark because it did not complete within the predefined runtime limit. Runtime is shown in minutes and memory usage is shown in GB.

13. Runtime Benchmarking Environment and Resource Allocation

Runtime benchmarking was conducted on a Slurm-managed Linux computing cluster. Most CPU-based methods were benchmarked on the CPU node cnode02, which was equipped with four Intel Xeon Platinum 8260 processors (2.40 GHz), each with 24 cores, providing 96 CPU cores in total, one thread per core, and 2 TB RAM. No GPU resources were available on this node.

scBC was run on the GPU node gnode02 because its model training requires GPU acceleration. This node was equipped with four Intel Xeon Platinum 8260 processors (2.40 GHz), providing 96 CPU cores in total, 740 GB RAM, and 6 NVIDIA A100-PCIE-40GB GPUs, each with 40,960 MiB of memory. Runtime for scBC was therefore measured using this GPU configuration, whereas the remaining CPU-based methods were benchmarked on the CPU node.

14. Algorithm Implementations and Software Environment

The compared algorithms were implemented in different programming languages and software ecosystems, including C++, R, Python, and Java. Therefore, each method was run using its official or recommended implementation in its native software environment. To improve reproducibility and transparency, the implementation source, programming language or platform, and key software dependencies for each method are summarized in Table S4. CPU-based methods were benchmarked on CPU compute nodes, whereas scBC was run on a GPU compute node because its model training relies on GPU acceleration.

Table S4. Implementation sources and software environments of the compared biclustering methods. The table summarizes the implementation source, programming language or platform, runtime environment, and key software dependencies used for each method.

Method	Implementation used	Language	Runtime environment / key dependencies	Hardware note
NoiBic	Compiled command-line implementation	C++	GCC/G++ 9.3.0; GNU Make 3.82	CPU node
RecBic	Compiled command-line implementation	C++	GCC/G++ 9.3.0; GNU Make 3.82	CPU node
ARBic	Compiled command-line implementation	C++	GCC/G++ 9.3.0; GNU Make 3.82	CPU node
QUBIC2	Compiled command-line implementation	C++	GCC/G++ 9.3.0; GNU Make 3.82	CPU node
RUBIC	Official GitHub implementation	C++ executable + Python post-processing	GCC/G++ 9.3.0; GNU Make 3.82 Python 3.7.9;	CPU node
QUBIC	R package QUBIC	R	QUBIC 1.30.0	CPU node
FABIA	R package fabia	R	fabia 2.48.0	CPU node
ISA	R package isa2	R	isa2 0.3.6	CPU node
UniBic	R package runibic	R	runibic 1.24.0	CPU node
CPB	Official CPB source package with Python wrapper run_cpb.py and compiled C executables cpb and init_bicluster	Python + C/C++	Python 2.7.18 BiBench 0.1; compiled CPB executables added to PATH	CPU node
OPSM	Command-line OPSM implementation provided as OPSM.jar and invoked through opsm.sh	Java + shell	Java runtime; OPSM_HOME set to OPSM installation directory; Python 2.7.18 BiBench 0.1;	CPU node
HBiC	Python package py_hbic	Python	Python 3.10.20; py_hbic 1.0;	CPU node
scBC	Python package scBC	Python	Python 3.10.18; scBC 0.3.2;	GPU node, NVIDIA A100

15. Overall statistical comparisons on single-cell RNA-seq datasets

To evaluate whether NoiBic consistently outperformed the competing methods across the nine annotated scRNA-seq datasets and tested parameter settings, we performed one-sided Wilcoxon rank-sum tests comparing NoiBic with each competing method separately for ARI and NMI. P-values were adjusted using the Benjamini-Hochberg procedure. The adjusted P-values were below 0.05 for all comparisons, supporting a significant overall advantage of NoiBic (Fig. S7).

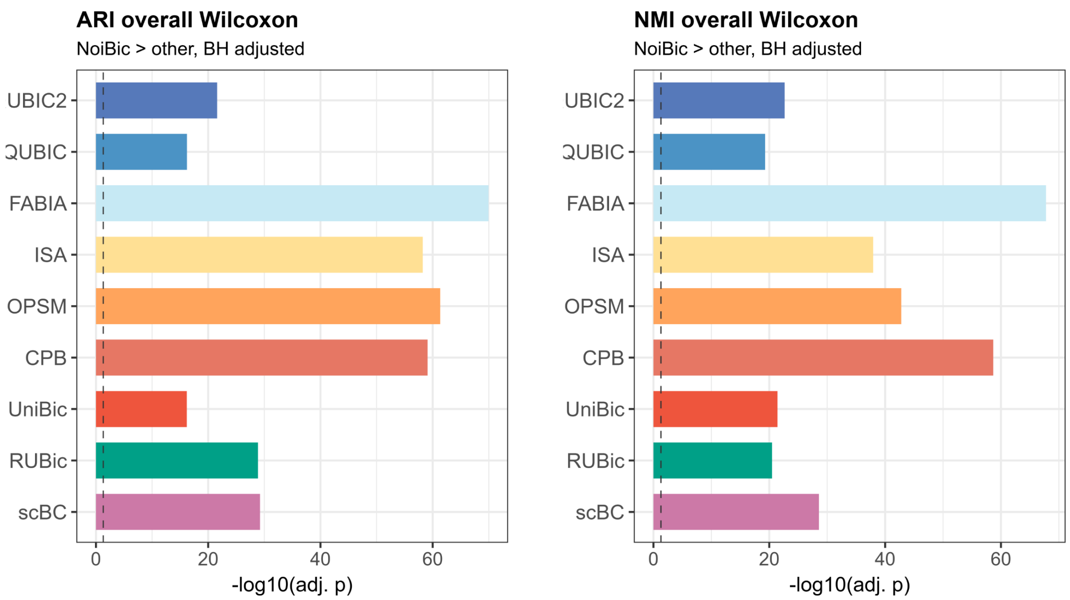

Fig. S7. Overall Wilcoxon rank-sum comparisons of NoiBic with competing methods on annotated scRNA-seq datasets. The left and right panels show comparisons based on ARI and NMI, respectively. Bars represent -log10 Benjamini-Hochberg-adjusted P-values from one-sided tests of whether NoiBic achieved higher scores than each competing method. The dashed line indicates an adjusted P-value of 0.05.

16. Volcano plots of human pleomorphic adenoma data constructed using the marker genes identified in the cells of biclusters.

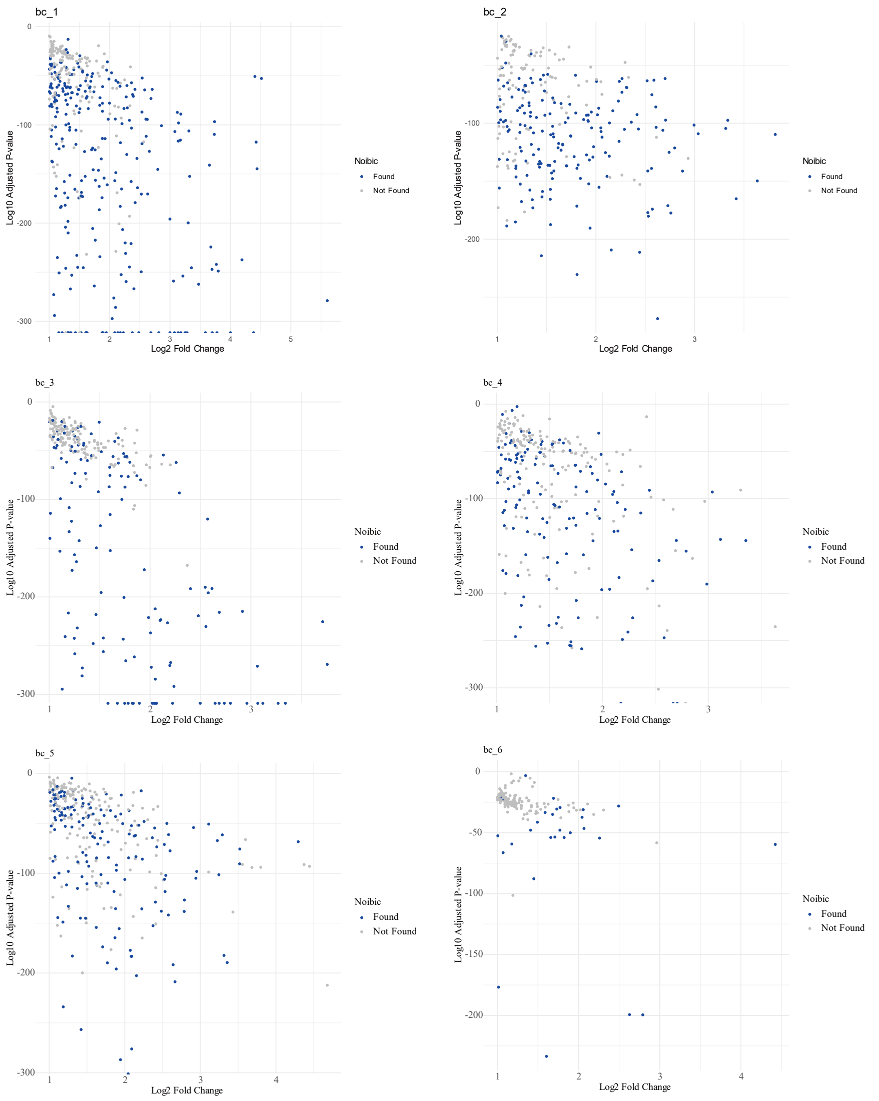

Fig. S8. Marker-gene analysis of NoiBic-identified biclusters in human pleomorphic adenoma data. Volcano plots show marker-gene statistics for the six biclusters. Genes included in the corresponding NoiBic bicluster are shown in blue, whereas the remaining genes are shown in grey.

17. Volcano plots of mouse embryonic stem cells data constructed using the marker genes identified in the cells of biclusters.

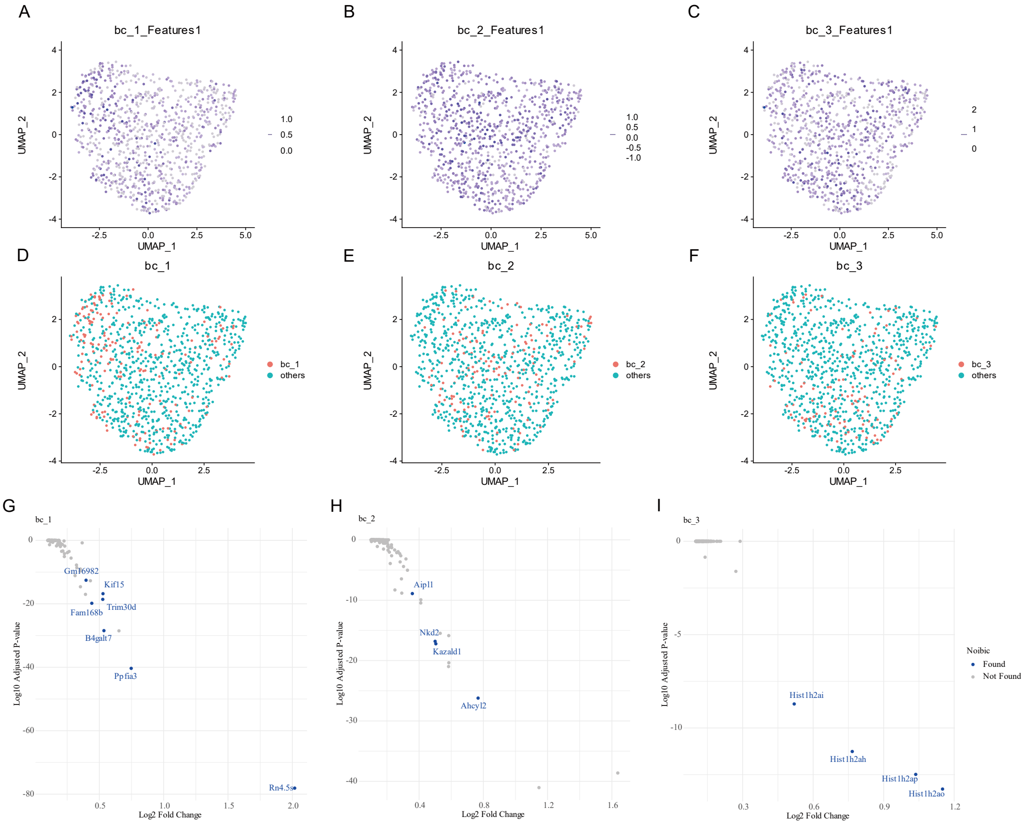

Fig. S9. Module and marker-gene characterization of NoiBic-identified biclusters in mouse embryonic stem cell data. (A-C) UMAP feature plots showing module scores for biclusters 1-3, respectively. (D-F) UMAP visualizations highlighting cells assigned to biclusters 1-3, respectively. (G-I) Volcano plots showing marker-gene statistics for biclusters 1-3, respectively. Genes included in the corresponding NoiBic bicluster are shown in blue, whereas the remaining genes are shown in grey. Selected representative genes are labelled, including histone H2A family genes in bicluster 3.
