# Supplementary material for: NoiBic: a noise-tolerant biclustering algorithm for high-throughput gene expression data analysis
Source: Bioinformatics. 2026 Oct 7;42(10):btag743. doi: 10.1093/bioinformatics/btag743 (PMC13681661; doi:10.1093/bioinformatics/btag743)
Supplement: Supplementary file 1 [file btag743_supplementary_data.zip › Supplementary File 2 Pseudocode of NoiBic.pdf]

Notation. Let $E \in \mathbb{R}^{n\times m}$ be the expression matrix, where rows denote genes and columns denote samples or cells. Let $\Gamma \in \mathbb{R}^{n\times m}$ be an optional entry-wise noise matrix; if it is not provided, NoiBic estimates the tolerated fluctuation from row-wise variation. For each row i, let

$$A_{i\cdot} = (A_{i,1}, A_{i,2}, \ldots)$$

be the sequence of original column indices sorted by the corresponding expression values in row i; thus $E_{i,A_{i,1}} \leq E_{i,A_{i,2}} \leq \cdots$ after missing values are removed. A bicluster is denoted by $E_{R,C}$, where R and C are row and column sets. In the implementation, R is represented as $R^+ \cup R^-$, where R^+ and R^- contain positively and negatively correlated rows, respectively. Parameters follow the manuscript notation: α for LACS exchange tolerance, δ for the minimum retained seed-length ratio in initial clustering, γ for column insertion tolerance, η for row/column expansion support, and ϵ for overlap filtering.

Algorithm 1: NoiBic

Input : Expression matrix E, optional noise matrix Γ, and parameters $\alpha, \delta, \gamma, \eta, \epsilon$

Output: Ranked biclusters $\mathcal{B}^*$

$A \leftarrow$ `BuildSortedIndex`(E) `// row-wise sorted column-index sequences`

if Γ *is not provided* **then**

  Estimate Γ from row-wise variation in E

$\mathcal{S} \leftarrow$ `SeedSelection`(E, A, Γ, α)

$\mathcal{B} \leftarrow$ `InitialBiclustering`$(E, A, \Gamma, \mathcal{S}, \alpha, \delta)$

if *column expansion is enabled* **then**

  `ColumnExpansion`$(E, \Gamma, \mathcal{B}, \gamma, \eta)$

if *row expansion is enabled* **then**

  `RowExpansion`$(E, A, \Gamma, \mathcal{B}, \alpha, \eta)$

$\mathcal{B}^* \leftarrow$ `PruneAndRank`$(\mathcal{B}, \epsilon)$

return the top requested biclusters in $\mathcal{B}^*$

Algorithm 2: Noise-aware LACS

Input : Two sorted index sequences $A_{u\cdot}$ and $A_{v\cdot}$, row indices u, v, expression matrix E, noise matrix Γ, tolerance α, and flag `storePath`

Output: (ℓ, σ, P), where ℓ is the LACS length, $\sigma = 1$ denotes positive correlation, $\sigma = 0$ denotes negative correlation, and P is the matched column path

Remove trailing missing entries from both sorted rows; let the remaining lengths be n_u and n_v

if *n_u or n_v is below the minimum column width* **then**
    return $(0, 1, \emptyset)$

Construct two orientations for row v: the increasing order $A^{+}_{v\cdot}$ and the decreasing order $A^{-}_{v\cdot}$

Initialize mutable copies of $A_{u\cdot}$ and $A^{s}_{v\cdot}$, index maps, DP tables D^s, and optional path tables H^s for $s \in \{+, -\}$

Compute repeat and constant-row flags for rows u and v using `RepeatStats`

foreach $s \in \{+, -\}$ **do**
    for $i \leftarrow 1$ **to** n_u **do**
        for $j \leftarrow 1$ **to** n_v **do**
            $p \leftarrow A_{u,i}$; $q \leftarrow A^{s}_{v,j}$
            if `ApproxMatch`(p, q, s, u, v) *succeeds* **then**
                $D^{s}_{i,j} \leftarrow D^{s}_{i-1,j-1} + 1$; record a diagonal step in H^s
            else if $D^{s}_{i-1,j} > D^{s}_{i,j-1}$ **then**
                $D^{s}_{i,j} \leftarrow D^{s}_{i-1,j}$; record an upward step in H^s
            else
                $D^{s}_{i,j} \leftarrow D^{s}_{i,j-1}$; record a leftward step in H^s

if $D^{+}_{n_u,n_v} \geq D^{-}_{n_u,n_v}$ **then**
    $\ell \leftarrow D^{+}_{n_u,n_v}$; $\sigma \leftarrow 1$
else
    $\ell \leftarrow D^{-}_{n_u,n_v}$; $\sigma \leftarrow 0$

$P \leftarrow$ `Traceback`$(H^{+/-})$ if `storePath` is true; otherwise $P \leftarrow \emptyset$

return (ℓ, σ, P)

Function `ApproxMatch`(p, q, s, u, v)**:**
    if $p = q$ **then**
        return success
    Let i' be the current position of column q in row u, and j' the current position of column p in row v
    if *either position is missing, or the two positions do not cross forward* **then**
        return failure
    $c_v \leftarrow$ `FeasibleExchange`(v, j, j', s); $c_u \leftarrow$ `FeasibleExchange`(u, i, i', s)
    if $c_u = c_v = -1$ **then**
        return failure
    Swap the feasible side with smaller exchange cost; if only one side is feasible, swap that side
    Update the corresponding mutable order and index map
    return success

Function `FeasibleExchange`(*row r, positions a, b, orientation s*)**:**
    Let p and q be the two column indices at positions a and b
    Reject the exchange if either column is absent, $b \leq a$, or row r fails the constant/repetition checks

Algorithm 3: Seed Selection

Input : Expression matrix E, sorted index matrix A, noise matrix Γ, LACS tolerance α, seed number K, and seed selection mode

Output: Priority queue $\mathcal{S}$

Cache repeat and constant-row flags for all rows
Partition the rows into four contiguous groups
$\mathcal{C} \leftarrow \emptyset$
foreach *group* G **do**
  foreach *row pair* (u, v) *in* G **do**
    $(\ell, \sigma, \emptyset) \leftarrow$ `LACS`$(A_{u\cdot}, A_{v\cdot}, u, v, E, \Gamma, \alpha,$ `false`$)$ `// rolling-array implementation`
    if ℓ *is below the minimum column width* **then**
      continue
    Add (u, v, ℓ, σ) to $\mathcal{C}$
if *mode keeps top seeds* **then**
  $\mathcal{S} \leftarrow$ the top K seeds by ℓ
else if *mode uses row-diverse selection* **then**
  Sort $\mathcal{C}$ by decreasing ℓ and select up to K seeds under row-count caps and an anchor gap n/K
else if *mode uses random selection* **then**
  $\mathcal{S} \leftarrow$ a random sample of up to K qualified seeds
else
  $\mathcal{S} \leftarrow \mathcal{C}$ `// all qualified seeds`
return $\mathcal{S}$

Algorithm 4: Initial Bicluster Identification

Input : Expression matrix E, sorted index matrix A, noise matrix Γ, seed queue $\mathcal{S}$, and parameters α, δ

Output: Initial bicluster set $\mathcal{B}$

$\mathcal{B} \leftarrow \emptyset$
while $\mathcal{S}$ *is not empty* **do**
    Pop the highest-ranked seed $S = (u, v, L, \sigma)$
    if $\neg$`SeedValid`$(S, \mathcal{B})$ **then**
        continue
    $(L, \sigma, P) \leftarrow$ `LACS`$(A_{u\cdot}, A_{v\cdot}, u, v, E, \Gamma, \alpha,$ `true`$)$
    Choose the less repetitive seed row as anchor a and order P by that row
    Initialize $C \leftarrow P$, $R^{+} \leftarrow \{a\}$, and $R^{-} \leftarrow \emptyset$
    Add the other seed row to R^{+} if $\sigma = 1$, otherwise to R^{-}
    Let $R = R^{+} \cup R^{-}$ and build the anchor sequence from row a on columns C
    foreach *row* $x \notin R$ **do**
        Compute $(\ell_x, \sigma_x, P_x) \leftarrow$ `LACS`$(A_{a,C}, A_{x,C}, a, x, E, \Gamma, \alpha,$ `true`$)$
    Rank candidate rows by decreasing ℓ_x
    while *the best candidate* x *satisfies* `GrowthOK(`$|C|, |R|, \ell_x, L, \delta$`)` **do**
        Add x to R^{+} if $\sigma_x = 1$, otherwise to R^{-}
        $C \leftarrow C \cap P_x$ and $R \leftarrow R^{+} \cup R^{-}$
        Intersect each remaining P_y with P_x, update ℓ_y, and re-rank candidates
    Add $E_{R,C}$ with row partition (R^{+}, R^{-}) to $\mathcal{B}$
return $\mathcal{B}$

`// GrowthOK implements the code condition:` ℓ_x `must exceed the absolute column-width threshold and` $L\delta$`, and adding the row must not decrease the smaller bicluster dimension; a special single-cell branch permits wide enough column support.`

Algorithm 5: Column Expansion by Insertion-slot Voting

Input : Biclusters $\mathcal{B}$, expression matrix E, noise matrix Γ, parameters γ, η
Output: Biclusters with expanded column sets

foreach *bicluster* $E_{R,C}$ *with row partition* (R^+, R^-) **do**
  if $|C|$ *or* $|R|$ *is below half the minimum column width* **then**
    continue
  Rebuild the current column set by testing each existing column with `InsertionVote` and retaining it only if its vote is at least $\eta|R|$
  Rank every nonmember column by increasing CV over rows R
  foreach *candidate column* $c' \notin C$ *in ranked order* **do**
    $s \leftarrow$ `InsertionVote`$(E_{R,C}, c')$
    if $s \geq \eta|R|$ **then**
      Insert c' into C
return $\mathcal{B}$

Function `InsertionVote`$(E_{R,C}, c')$**:**
  Initialize slot counts $V_0, \ldots, V_{|C|} \leftarrow 0$ and $s \leftarrow 0$
  foreach $r \in R^-$ **do**
    `Vote`$(r, c', C, \text{decreasing}, V, s)$
  foreach $r \in R^+$ **do**
    `Vote`$(r, c', C, \text{increasing}, V, s)$
  return s

Function `Vote`$(r, c', C, \text{order}, V, s)$**:**
  if $E_{r,c'}$ *is missing* **then**
    return
  Sort the non-missing values $\{E_{r,c} : c \in C\}$ by *order*
  $\Delta \leftarrow \Gamma_{r,c'}\gamma$
  $I \leftarrow [\min(E_{r,c'}(1-\Delta), E_{r,c'}(1+\Delta)), \max(E_{r,c'}(1-\Delta), E_{r,c'}(1+\Delta))]$
  Find all insertion slots whose value range is compatible with I by binary search
  Increment those slots and update s to the largest slot count

Algorithm 6: Row Expansion

Input : Biclusters $\mathcal{B}$, expression matrix E, sorted index matrix A, noise matrix Γ, parameters α, η

Output: Biclusters with expanded row sets

foreach *original bicluster* $E_{R,C}$ *with seed anchors* u, v **do**
- **if** $|C|$ *is below half the minimum column width* **then**
  - **continue**
- Build sorted anchor sequences from rows u and v restricted to C
- **if** *the second anchor is not highly repetitive* **then**
  - Create a mirror bicluster by swapping the two anchors and exchanging R^+, R^- when $\sigma = 0$
- **foreach** *row* $x \notin R$ **do**
  - $(\ell_x, \tau_x, \emptyset) \leftarrow \texttt{LACS}(A_{u,C}, A_{x,C}, u, x, E, \Gamma, \alpha, \texttt{false})$
  - **if** $\ell_x \geq \eta|C|$ **then**
    - Add x to R^+ if $\tau_x = 1$, otherwise to R^-
  - **if** *a mirror bicluster exists* **then**
    - $(\ell'_x, \tau'_x, \emptyset) \leftarrow \texttt{LACS}(A_{v,C}, A_{x,C}, v, x, E, \Gamma, \alpha, \texttt{false})$
    - **if** $\ell'_x \geq \eta|C|$ **then**
      - Add x to the corresponding row set of the mirror bicluster
- Keep both valid biclusters; if only the mirror is valid, replace the original by the mirror

return $\mathcal{B}$

Algorithm 7: Overlap Pruning and Ranking

Input : Expanded biclusters $\mathcal{B}$ and overlap threshold ϵ

Output: Filtered and ranked biclusters $\mathcal{B}^*$

Set the area of each $E_{R,C}$ to $|R||C|$

foreach *pair* E_{R_1,C_1}, E_{R_2,C_2} **do**
- $\rho_R \leftarrow |R_1 \cap R_2| / \min(|R_1|, |R_2|)$
- $\rho_C \leftarrow |C_1 \cap C_2| / \min(|C_1|, |C_2|)$
- **if** $\rho_R \geq \epsilon$ *and* $\rho_C \geq \epsilon$ **then**
  - Replace the smaller-area bicluster by its non-overlapping residual rows and columns

Sort biclusters by decreasing area, breaking ties by row count

Discard biclusters that fail the row-width, column-width, minimum-size, or single-cell width constraints

return the remaining biclusters
